# Serological Immunoglobulin-Free Light Chain Profile in Myasthenia Gravis Patients

**DOI:** 10.1155/2018/9646209

**Published:** 2018-03-25

**Authors:** Umberto Basile, Mariapaola Marino, Cecilia Napodano, Krizia Pocino, Paolo Emilio Alboini, Francesca Gulli, Amelia Evoli, Carlo Provenzano, Emanuela Bartoccioni

**Affiliations:** ^1^Dipartimento di Medicina di Laboratorio, Fondazione Policlinico Universitario Agostino Gemelli, Università Cattolica del Sacro Cuore, Rome, Italy; ^2^Istituto di Patologia Generale, Fondazione Policlinico Universitario Agostino Gemelli, Università Cattolica del Sacro Cuore, Rome, Italy; ^3^Istituto di Neurologia, Dipartimento di Geriatria, Neuroscienze e Ortopedia, Fondazione Policlinico Universitario Agostino Gemelli, Università Cattolica del Sacro Cuore, Rome, Italy; ^4^Dipartimento di Medicina di Laboratorio, Ospedale Madre Giuseppina Vannini, Rome, Italy

## Abstract

**Background:**

Serological levels of free immunoglobulin light chains (FLCs), produced in excess of heavy chains during synthesis of immunoglobulins by plasma cells, can be considered a direct marker of B cell activity in different systemic inflammatory-autoimmune conditions and may represent a useful predictor of rituximab (RTX) therapeutic efficacy, as reported for rheumatoid arthritis and systemic lupus erythematosus. Myasthenia gravis (MG) is an autoimmune disease of the neuromuscular junction with antibodies (abs) targeting the acetylcholine receptor (AChR) or the muscle-specific tyrosine kinase (MuSK), inducing muscle weakness and excessive fatigability. As MG course may be remarkably variable, we evaluated the possible use of FLCs as biomarkers of disease activity.

**Subjects and Methods:**

We assessed FLC levels in 34 sera from 17 AChR-MG and from 13 MuSK-MG patients, in comparison with 20 sera from patients with systemic autoimmune rheumatic diseases and 18 from healthy blood donors, along with titers of specific auto-abs and IgG subclass distribution.

**Results:**

We found a statistically significant increase in free *κ* chains in both AChR- and MuSK-MG patients, while free *λ* chain levels were increased only in AChR-MG. We also observed a significant reduction of both free *κ* and *λ* chains in 1/4 MuSK-MG patients along with specific abs titer, two months after RTX treatment.

**Conclusions:**

From our data, FLCs appear to be a sensitive marker of B cell activation in MG. Further investigations are necessary to exploit their potential as reliable biomarkers of disease activity.

## 1. Introduction

Activation of autoreactive B lymphocytes, leading to their differentiation into autoantibodies (auto-abs) producing plasma cells, is the most important pathogenetic mechanism in several autoimmune diseases. Immunoglobulin- (Ig-) free light chains (FLCs) are produced in excess of heavy chains during the synthesis of intact Ig by plasma cells [1] and contribute to inflammation in experimental disease models [2]. In serum, these excess polyclonal FLCs have a short half-life (2–6 hours), and they are excreted by the kidney [3]. Therefore, an increase in their circulating levels reflects either a decreased clearance because of kidney failure [4] or an increased production. Thanks to their short half-life, and in subjects with normal kidney function, their serum levels can be considered as a direct marker of B cell activity, which is otherwise difficult to measure in routine clinical practice. As a matter of fact, the quantitative assay of *κ* and *λ* FLCs and the *κ*/*λ* ratio is a useful diagnostic tool in plasma cell dyscrasias, such as mixed cryoglobulinemia, multiple myeloma, monoclonal gammopathy of undetermined significance, and amyloidosis [5–7]. In the last few years, elevated concentrations of polyclonal FLCs in the serum and urine have been reported in patients with rheumatoid arthritis (RA), systemic sclerosis (SS), primary Sjogren syndrome (pSS), and systemic lupus erythematosus (SLE) [8–11]. Serum FLCs have no significant antigen-binding activity and, therefore, are not consumed in immune-inflammatory reactions unlike other molecules (complement, immune complexes, Ig, and auto-abs) that are used as biomarkers of disease activity. Because of these characteristics, they may outperform more widely used biomarkers in evaluating disease activity and predict flares in RA and SLE patients [2, 9].

Myasthenia gravis (MG) is a rare autoimmune disorder with an incidence estimated to be 1-2 per 100,000 and a prevalence of 7–20 per 100,000 [12]. Auto-abs bind to well-defined antigens in the postsynaptic membrane at the neuromuscular junction and impair nerve-muscle transmission, which in turn induces muscle weakness and excessive fatigability. In approximately 85% of the patients, abs are directed against the nicotinic acetylcholine receptors (AChR) [13] while a smaller portion of MG patients produce abs against the muscle specific tyrosine kinase (MuSK) [14, 15] or the low-density lipoprotein receptor-related protein 4 [16]. Along with age at onset and thymus pathology, the auto- abs status is used in the definition of disease subgroups [17].

Due to the fluctuating nature and heterogeneity of the disease, the diagnosis of MG can be puzzling. It is confirmed by the combination of typical symptoms and signs, electromyographic and pharmacological tests, as well as by the detection of specific auto-abs. Disease management can be difficult: therapy must be tailored on the single patient, but the clinical course may be unpredictable and the therapeutic response is highly variable. In order to find a measure of disease activity, many groups focused on the analysis of proinflammatory and anti-inflammatory cytokines and molecules: many of them showed significant differences between patients and controls [18–20] but there is no validated commercial kit to routinely measure them. Also, changes of T and B cell subsets were evaluated as possible biomarkers of disease activity and eventually as therapeutic targets [21].

FLCs have never been investigated in MG but could be useful to predict possible variations of B cell activity, which can influence the clinical picture both in the short and in the long range. In this study, we explored the levels of FLCs in MG patients, along with titers of specific auto-abs and IgG subclasses. These results were compared with those from patients with systemic autoimmune rheumatic diseases (SARD) and from healthy blood donors (HBD).

## 2. Materials and Methods

### 2.1. Patients and Controls

We collected 34 sera from 30 MG patients referred to the Department of Neurosciences of the Fondazione Policlinico Agostino Gemelli in Rome from June 2007 to April 2016. All patients had generalized MG, as defined by the Myasthenia Gravis Foundation of America (MGFA) classification [22]. MG patients were treated according to the accepted guidelines [23] and are detailed in Supplementary Tables 1 and 2. Seventeen patients had AChR-abs and 13 had anti-MuSK abs; in 4 MuSK-MG patients, a second sample was collected after rituximab (RTX) treatment.

The SARD control group included 12 SLE patients (positive for antinuclear abs with homogeneous pattern and with high levels of anti-double-stranded DNA abs) and 8 RA patients (positive for antinuclear abs with coarse speckled pattern and with high levels of anticyclic citrullinated peptide abs) naïve to drug treatment. Sera from 18 HBD were used as negative controls.

Five MuSK-MG patients (#1, #10, #11, #12, and #13) received RTX at a dose of 375 mg/m2 once a week for 4 consecutive weeks [24]. Efficacy of RTX treatment was verified by cytofluorimetric CD19+ cell count before and after treatment.

All patients had an estimated glomerular filtration rate (eGFR) ≥ 60 mL/min/1.73 m^2^. None of them underwent plasmapheresis, nor received high dose intravenous immunoglobulins, during this study. The whole study was conducted according to the Declaration of Helsinki and approved by the Ethical Committee of the Università Cattolica; all the participants provided written informed consent before enrollment. All samples were processed anonymously.

### 2.2. Laboratory Testing

Sera were obtained by standard centrifugation, divided into aliquots, and stored frozen until analysis. Samples were thawed only once and immediately assayed in a blinded fashion and in a single batch.

FLCs were assessed using the Freelite™ Human Kappa and Lambda Free Kits (The Binding Site, Birmingham, UK) on a SPAPLUS instrument (The Binding Site, UK; free *κ* normal range: 3.3–19.4 mg/L; free *λ* normal range: 5.7–26.3 mg/L). A ratio of *κ*/*λ* < 0.26 or >1.65 is abnormal, according to the manufacturer's recommendations.

The four IgG subclasses concentration were measured by turbidimetry (Human IgG and IgG subclass liquid reagent kits, The Binding Site) on the SPAPLUS instrument according to the manufacturer's recommendations. Normal range for subclasses: 3.82–9.29 g/L for IgG1; 2.42–7.0 g/L for IgG2; 0.22–1.76 g/L for IgG3; and 0.04–0.86 g/L for IgG4.

Anti-AChR and Anti-MuSK antibodies were detected by radioimmunoprecipitation assay using, respectively, AChR-Ab RIA Kit (cut-off ≥ 0.5 nmol/L) and MuSK-Ab RIA Kit (RSR Cardiff, UK) (cut-off ≥ 0.05 nmol/L) according to the manufacturer's instructions.

### 2.3. Statistical Analysis

Comparison of mean values was performed by Student's *t*-test; *p* values < 0.05 were considered significant. A correlation analysis was carried out using the Pearson correlation coefficient.

## 3. Results

### 3.1. Serum FLC Assessment in Patients and Controls

Determinations of antibody titer, free *κ*, free *λ*, *κ*/*λ* ratio, and IgG subclasses in all samples from AChR- and MuSK-MG patients are reported in Supplementary Tables 1 and 2, respectively. In AChR-MG patients, 11 and 4 out of 17 samples had a free *κ* or *λ* value, respectively, above the range of normality, while 1/17 had a *λ* value below the range of normality; in MuSK-MG patients, 8 out of the 13 samples displayed a free *κ* value above the range of normality while 2/13 had a value of free *λ* above and 2/13 below the range of normality.

The mean values (±standard deviation) of FCLs and *κ*/*λ* ratio are reported in Table 1. The statistical analysis revealed significant differences between patients and HBD: the mean values of free *κ* were above the cut-off and significantly higher in both MG subgroups and in SARD patients as compared to HBD. The mean value of *λ*-free chain levels was significantly different only in AChR-MG patients when compared to HBD, even if it was still within the normal value (as determined by the manufacturer); SARD patients had higher *λ* levels both when compared to MG patients and to HBD, but differences were not statistically significant because of the high standard deviation. The *κ*/*λ* ratio was significantly higher in both MG subgroups and in the SARD group when compared to HBD.

### 3.2. Correlation between Serum FLC Levels and Antibody Titer in MG Patients

**Figure 1:**
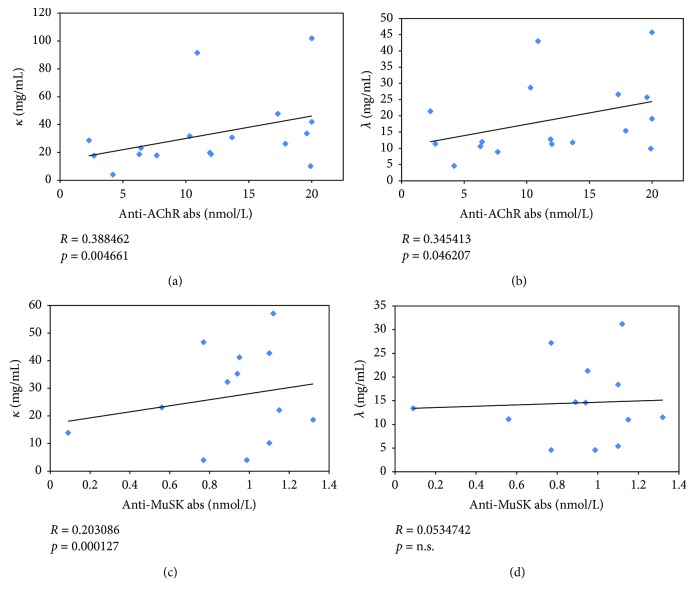
Correlation between *κ* and *λ* levels and anti-AChR (a and b) and anti-MuSK (c and d) antibodies.

We evaluated if levels of serum FLCs in MG patients correlated with the specific antibody titer. We found that both free *κ* and *λ* chains had a moderate, but significant, correlation with anti-AChR abs titer (*R* = 0.388462, *p* = 0.004661 for *κ*; *R* = 0.345413, *p* = 0.046207 for *λ*), while only free *κ* chains had a weak correlation with anti-MuSK abs titer (*R* = 0.203086, *p* = 0.000127) (Figure 1).

### 3.3. Serum IgG Subclass Levels among Patients and Controls

**Figure 2:**
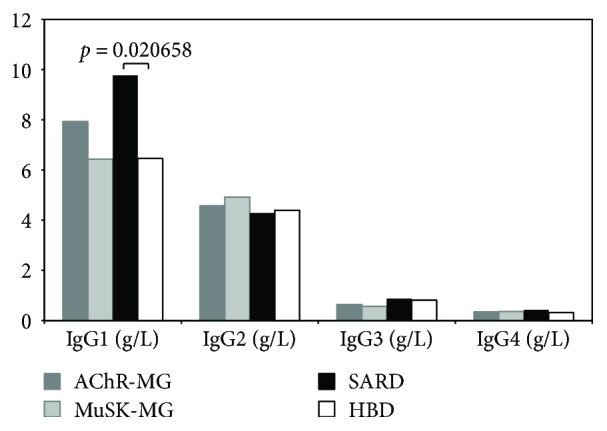
Serum IgG subclass levels in AChR-MG, MuSK-MG, SARD, and HBD.

**Figure 3:**
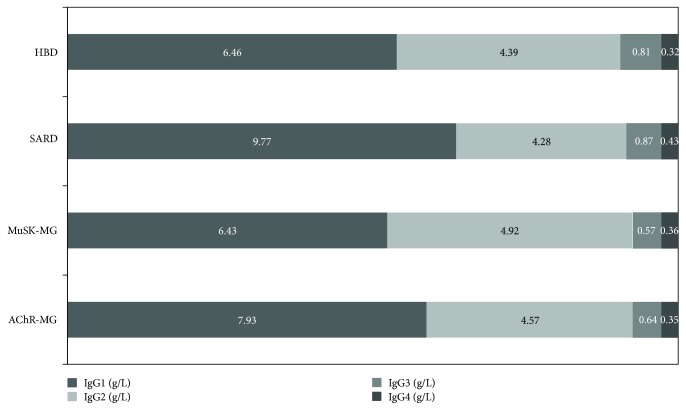
Serum IgG1–4/IgG distribution in HBD, SARD, MuSK-MG, and AChR-MG.

The mean values (± standard deviation) of IgG subclasses are reported in Table 2 and visualized in Figures 2 and 3. Only serum IgG1 levels in SARD patients displayed a mean value (9.77 ± 5.68 g/L) which was above the normal range (3.82–9.29 g/L) and statistically different from our HBD mean value (6.46 ± 1.64 g/L, *p* = 0.020658).

### 3.4. Serological Parameters in Rituximab-Treated MuSK-MG Patients

We were able to measure retrospectively specific auto-abs and FLCs in two blood samples of 4 MuSK-MG patients treated with rituximab (#10–13, Supplementary Table 2), collected at different time points before first infusion (8–60 months) and 3–8 weeks after first RTX treatment (Table 3).

Cytofluorimetric CD19+ cell count displayed a reduction greater than 90% after RTX in all 4 patients. As shown in Table 3, only patient #13 displayed a strong decrease in all serological parameters, along with MGFA score, 8 weeks after the first infusion of RTX while the other 3/4 patients, who were examined at a shorter interval, had variable, but moderate, variations.

## 4. Discussion

Quantitative analysis of immunoglobulin chain synthesis by B cells demonstrated that there is an excess of light chain production [1] which are then released in the general circulation. Being a by-product of intact immunoglobulin synthesis, they may represent a marker of overall B cell activity, particularly in those diseases where there is an increased formation of immune complexes like systemic autoimmune disorders. In these conditions, characterized by chronic inflammatory reactions, FLCs may play a pathogenetic role thanks to their enzymatic activities and binding to intra- and extracellular proteins, which, in turn, can initiate and maintain the inflammatory cascade [25]. Previous reports confirmed that patients with systemic autoimmune diseases have FLC levels significantly higher than the normal population, with a normal *κ*/*λ* ratio. These studies focused on relatively common SARD, in particular, SLE, RA, and SS: concentrations of FLCs were found to be significantly increased and have been investigated as possible biomarkers of the progression and severity of these chronic inflammatory diseases [8–11] as well as a potential therapeutic target [25]. In these conditions, the normality of *κ*/*λ* ratio, while free *κ* and *λ* chains were elevated, has been explained with their polyclonal production.

Little, if nothing, is known about FLC levels in organ-specific autoimmune diseases. Here, we report our results on FLC analysis in MG patients with anti-AChR and anti-MuSK auto-abs, and compare them with HBD and SARD patients. Our data demonstrate a statistically significant increase in free *κ* chains in both AChR- and MuSK-MG, as well as in SARD, when compared to HBD, with mean values more elevated in SARD than in MG (Table 1). When we analyzed free *λ* chain levels, we found that only in AChR-MG there was a little but statistically significant increase if compared to HBD, while they were normal in MuSK-MG: this result could be ascribed to the different immunosuppressive therapies that have been used in these two groups of patients. These differences in the trend of *κ* FLCs (definite increased in both MG subgroups) and *λ* FLCs (increased to less extents only in AChR-MG) can ultimately explain why we observe a statistically significant increase of the *κ*/*λ* ratio in both MG subgroups. SARD patients had greatly increased free *λ* levels, compared to both HBD and MG patients, but differences were not significant because of higher standard deviations (Table 1).

The finding of a correlation of anti-AChR abs with both *κ* and *λ* FLCs, as opposed to a weaker correlation of only *κ* FLCs with anti-MuSK abs, can be explained by the demonstrated different antibody repertoire in MuSK-MG as compared to AChR-MG [26].

Our finding of a small, but significant, increase of FLCs in MG may reflect the different pathogenetic mechanism between organ specific and systemic autoimmune diseases like SLE and RA: in the latter conditions, FLCs can increase the inflammatory reaction, which is a relevant pathogenic aspect of these diseases [25] that is not present in an organ-specific autoimmune disease like MG.

Rituximab emerged as an effective option in those MG patients refractory to conventional immunosuppression, with particular benefit for patients with MuSK-MG [27]. It is a chimeric mouse/human monoclonal antibody against the surface antigen CD20, which is expressed during early pre-B cell development: it is present on naïve and memory B cells, but not on stem cells or fully differentiated plasma cells. The human protein is part of a multimeric complex regulating Calcium transport across the cell membrane, thus controlling B lymphocytes activation and proliferation; accordingly, RTX binding to CD20 interferes with these processes [28]. Circulating Ig-producing long-lived plasma cells are not depleted by RTX [29]. A proposed model for MuSK-MG responsiveness to RTX suggests that the consistent reduction in MuSK-MG auto-abs titer, seen as early as 3 months after first infusion, depends on short-lived antibody-secreting plasmablasts. As only a small fraction of these cells is CD20+, MuSK-MG outcome may depend on depletion of a pool of plasmablast-progenitor CD20+ memory B cells; alternatively, the direct depletion of the CD20+ fraction of plasmablasts by rituximab could contribute to clinical response [30, 31]. B cells and plasmablasts seem to be key players in several autoimmune, RTX-responsive disorders [32]. Recent studies evaluated the role of FLCs as a marker of the therapeutic efficacy of RTX in patients with SLE and RA and their results seemed promising [33, 34]. To investigate if serum FLCs can be considered a biomarker of both disease activity and effectiveness of RTX treatment, we retrospectively analyzed the serological profile in 4 (#10–13) out of our 13 MuSK-MG patients, before and 3–8 weeks after RTX infusion, when CD19+ cell count was decreased by more than 90%. We observed a significant reduction of both free *κ* and *λ* chains (−58.20% and −48.98%, resp.) only in patient #13, 8 weeks after the first RTX infusion. Even if it remained above the cut-off of positivity, also specific anti-MuSK auto-abs titer decreased from 0.89 to 0.49 nmol/L (−44.94%) along with a reduction of all IgG subclasses (Supplementary Table S2). In the other three RTX-treated patients, the post-RTX samples were drawn after a shorter interval (3–6 weeks), so that it was perhaps too early to observe the effect of CD20+ cells depletion, either on FLCs or on anti-MuSK abs titers. This finding is consistent with previous reports [30, 33]. On the whole, our results suggest that serum FLCs may represent a new marker of B cell activation in MG, which parallels auto-abs titer variations in response to B cell depleting therapy.

Most of the auto-abs are class G Ig (IgG), which includes 4 subclasses (IgG1–4). These IgG subclasses could contribute to the immunopathogenesis by modulating interaction of Ig, Fc-*γ* receptor and complement. Differences in serum IgG subclasses distribution between patients with autoimmune diseases and healthy controls have been described only recently, with distinct patterns in different conditions [35–37]. Here, we reported the IgG subclass mean levels and distribution among AChR-MG, MuSK-MG, SARD, and HBD (Table 2, Figures 2 and 3): only IgG1 in SARD had a statistically significant difference when compared to HBD and was above the range of normality. Our results do not suggest that IgG subclasses can have a diagnostic role in MG, and they further strengthen the hypothesis that serum IgG subclass distribution has peculiar characteristics in different autoimmune disease as already reported in a cross-sectional study in other autoimmune diseases [37]. Further elucidating these characteristics could lead to a better understanding of their pathogenetic roles in autoimmune disease development.

During the last twenty years, a novel systemic, chronic, and inflammatory disease entity with specific features has been described: IgG4-related disease (IgG4-RD) [38]. It is an uncommon immune-mediated inflammatory condition that affects a wide variety of organs, including the nervous system, characterized by tissue infiltration with IgG4+ plasma cells, storiform fibrosis, and frequent increase of serum IgG4 levels. Most anti-MuSK auto-abs belong to the IgG4 subclass [39], which cause MG in mice and humans by directly interfering with MuSK function [40, 41]. However, MuSK-MG does not fulfill IgG4-RD criteria, even if a recent case report opens the question of a possible link between the two diseases [42]. Our results did not show any significant increase in IgG4 mean values in all patients (AChR-, MuSK-MG, and SARD) when compared to HBD.

There is a great need for biomarkers in MG to identify patients at risk for disease flares, monitor response to treatment, and be a guide to a better management of immunosuppression. Different groups had tried to identify such markers: they analyzed large panels of proinflammatory and anti-inflammatory cytokines and molecules, finding many analytes which can be promising [18–20]. Several reports, analysing T and B cell subsets in MG patients, showed a disequilibrium between follicular and regulatory T cells together with a lower frequency of regulatory B cells. Longitudinal studies, evaluating changes in these cell subsets in different MG phases and in response to treatment, will clarify their role as markers of disease activity [21].

A truly useful biomarker should meet strict criteria: ideally, it should be central to the pathophysiology of the disease, it should be related to the disease activity and severity, it should fluctuate only with clinical flares or progression, and it should be sensitive to treatments known to be effective. We are conscious that there are limitations to our study. First, due to the small sample size, only a few potential confounders could be controlled, mainly those variables known to influence FLC levels, like kidney failure and plasmapheresis. Second, we could not include an active control group of subjects with acute viral and/or bacterial infections. Finally, our analytical method was consistent with previously reported studies [2, 9], but we acknowledge that it may not be the optimal one [43]. For these reasons, at the present time, we cannot suggest that the determination of FLCs has clinical relevance, with a cost/benefit ratio justifying its use in MG clinical management. Further investigations using longitudinal analysis will be necessary to validate it as a valuable tool to predict MG fluctuations and to monitor clinical management. Our goal, however, was to conduct a pilot study on serum levels of FLCs and IgG subclasses in an organ-specific autoimmune disease like MG. We report for the first time an increase of serum free *κ* chains in both AChR- and MuSK-MG patients, not correlated with an increase of circulating IgG: this can be explained with a continuous activation of the immune response which, as it is aimed at a specific autoantigen, does not reach levels as high as to increase total IgG. In this scenario, FLC determination seems to be a sensitive index of B cell activation even in organ-specific disorders where the autoimmune response is limited at a well-defined, single autoantigen.

## Figures and Tables

**Table 1:** *κ*, *λ*, and k/*λ* ratio mean values in patients and controls.

	*κ*-free mg/L	*λ*-free mg/L	*κ*/*λ*
AChR-MG	33.14 ± 26.29 (*p* < 0.05)	18.76 ± 11.79 (*p* < 0.05)	1.69 ± 0.47 (*p* < 0.05)
MuSK-MG	27.02 ± 16.98 (*p* < 0.05)	14.54 ± 8.24 (*p* n.s.)	1.75 ± 0.52 (*p* < 0.05)
SARD	71.65 ± 115.55 (*p* < 0.05)	52.14 ± 101.54 (*p* n.s.)	1.76 ± 0.73 (*p* < 0.05)
HBD	16.09 ± 4.28	11.82 ± 3.07	1.39 ± 0.28

Normal range for FLCs: 3.3–19.4 mg/L for *κ* and 5.7–26.3 mg/L for *λ*. A ratio of *κ*/*λ* < 0.26 or >1.65 is abnormal; *p* was calculated between each patient group and HBD. n.s.: not significant.

**Table 2:** IgG subclasses distribution in patients and controls.

	AChR-MG	MuSK-MG	SARD	HBD
IgG1, g/L	7.93 ± 5.73	6.43 ± 4.75	9.77 ± 5.68^∗^	6.46 ± 1.64
IgG2, g/L	4.57 ± 2.68	4.92 ± 2.90	4.28 ± 1.53	4.39 ± 1.06
IgG3, g/L	0.64 ± 0.45	0.57 ± 0.49	0.87 ± 0.60	0.81 ± 0.31
IgG4, g/L	0.35 ± 0.37	0.36 ± 0.34	0.43 ± 0.42	0.32 ± 0.19

Normal range for subclasses: 3.82–9.29 g/L for IgG1; 2.42–7.0 g/L for IgG2; 0.22–1.76 g/L for IgG3; and 0.04–0.86 g/L for IgG4. ^∗^ *p* = 0.02.

**Table 3:** Serological parameters in four MuSK-MG patients pre and post rituximab therapy.

Patients	RTX therapy	Anti-MuSK abs nmol/L	*κ*-free mg/mL	*λ*-free mg/mL	*κ*/*λ*	CD19+ cell count %	MGFA score
#10	Pre	0.77	4.00	4.60	0.87	9.40	III b
	Post 3 weeks	0.83	4.00	4.60	0.87	0.40	III b
Δ%		+7.79	0.00	0.00	0.00	−95.74	

#11	Pre	1.32	18.60	11.50	1.62	11.00	III b
	Post 4 weeks	1.41	27.60	14.90	1.85	0.10	II b
Δ%		+6.82	+48.39	+29.57	+14.2	−99.09	

#12	Pre	1.12	57.10	31.20	1.83	15.00	III b
	Post 6 weeks	1.09	49.80	41.10	1.21	1.00	III b
Δ%		−2.68	−12.78	+31.73	−33.88	−93.34	

#13	Pre	0.89	32.30	14.70	2.20	4.00	IV b
	Post 8 weeks	0.49	13.50	7.50	1.80	0.20	III b
Δ%		−44.94	−58.20	−48.98	−18.18	−95.00	

RTX therapy: the pretherapy sample was collected 8–60 months before first infusion. CD19+ cell count: percent over total peripheral blood lymphocytes. Δ% represents the percent variation over the pretherapy sample.

## Supplementary Materials

Supplementary 1 Table S1: the anonymous data set of AChR-MG patients.

Supplementary 2 Table S2: the anonymous data set of MuSK-MG patients.
