# Supplementary material for: Serological Immunoglobulin-Free Light Chain Profile in Myasthenia Gravis Patients
Source: J Immunol Res. 2018 Mar 25;2018:9646209. doi: 10.1155/2018/9646209 (PMC5889870; doi:10.1155/2018/9646209)
Supplement: Supplementary 1 — Table S1: the anonymous data set of AChR-MG patients. [file 9646209.f1.docx]

**Table S1. The anonymous data set of AChR-MG patients**

| **Pts** | **Age at onset** | **Gender** | **Anti-AChR abs**  **(nmol/L)** | **k free**  **(mg/L)** | **λ free**  **(mg/L)** | **k/λ** | **IgG1**  **(g/L)** | **IgG2**  **(g/L)** | **IgG3**  **(g/L)** | **IgG4**  **(g/L)** | **MGFA class at blood draw** | **Immunosuppressive**  **herapy** | **Dose of P**  **(mg)** |
| --- | --- | --- | --- | --- | --- | --- | --- | --- | --- | --- | --- | --- | --- |
| 1 | 28 | F | 20.00 | 101.90 | 45.70 | 2.23 | 12.40 | 10.20 | 1.47 | 0.44 | CSR | none |  |
| 2 | 53 | M | 17.9 | 26.20 | 15.40 | 1.70 | 3.56 | 2.23 | 0.62 | 0.19 | III b | P+ChE | 25 e/o/d |
| 3 | 69 | M | 6.30 | 18.60 | 10.60 | 1.75 | 3.64 | 2.87 | 0.95 | 0.09 | II a | P+A+ChE | 25 e/o/d |
| 4 | 65 | F | 20.00 | 41.90 | 19.10 | 2.19 | 9.29 | 6.07 | 0.60 | 1.10 | II a | P+A+ChE | 17 e/o/d |
| 5 | 38 | F | 10.90 | 91.50 | 43.00 | 2.13 | 25.23 | 9.03 | 1.50 | 1.20 | III a | ChE |  |
| 6 | 40 | F | 7.68 | 17.80 | 8.90 | 2.00 | 3.57 | 5.44 | 0.56 | 0.06 | II a | ChE |  |
| 7 | 74 | M | 17.30 | 47.70 | 26.60 | 1.79 | 12.10 | 4.99 | 0.56 | 0.21 | II b | P+A+ChE | 37.5 d |
| 8 | 62 | F | 13.66 | 30.70 | 11.80 | 2.60 | 8.91 | 3.27 | 1.01 | 0.17 | III b | P+A+Cy+ChE+RTX | 50 e/o/d |
| 9 | 40 | M | 12.00 | 18.70 | 11.30 | 1.65 | 4.66 | 5.21 | 0.43 | 0.24 | III a | P+A+ChE | 37.5 e/o/d |
| 10 | 39 | F | 4.20 | 4.00 | 4.60 | 0.87 | 1.58 | 0.20 | 0.06 | 0.03 | II a | ChE |  |
| 11 | 75 | F | 2.30 | 28.60 | 21.40 | 1.34 | 5.65 | 3.31 | 1.00 | 0.18 | II a | P | 50 d |
| 12 | 72 | M | 19.60 | 33.60 | 25.70 | 1.31 | 8.88 | 4.10 | 0.06 | 0.03 | III a | P+A+ChE | 50 d |
| 13 | 70 | M | 10.30 | 31.60 | 28.70 | 1.10 | 7.80 | 5.50 | 0.60 | 0.90 | III a | P+A+ChE | 50 d |
| 14 | 52 | F | 11.90 | 19.80 | 12.80 | 1.55 | 5.10 | 3.30 | 0.44 | 0.35 | III a | ChE |  |
| 15 | 59 | F | 19.90 | 10.10 | 9.90 | 1.02 | 1.58 | 0.20 | 0.06 | 0.03 | III b | P+ChE | 37.5 e/o/d |
| 16 | 18 | F | 2.70 | 17.60 | 11.40 | 1.54 | 8.00 | 4.50 | 0.90 | 0.32 | III a | ChE |  |
| 17 | 18 | F | 6.43 | 23.10 | 12.00 | 1.93 | 12.90 | 7.28 | 0.06 | 0.49 | IV b | P+ChE | 75 e/o/d |

Abbreviations: CSR=complete stable remission; P= prednisone; e/o/d= every other day; d= daily; CY= cyclosporine; ChE= acetylcholinesterase inhibitors; A= azathioprine; RTX=rituximab.
