# Supplementary material for: Serological Immunoglobulin-Free Light Chain Profile in Myasthenia Gravis Patients
Source: J Immunol Res. 2018 Mar 25;2018:9646209. doi: 10.1155/2018/9646209 (PMC5889870; doi:10.1155/2018/9646209)
Supplement: Supplementary 2 — Table S2: the anonymous data set of MuSK-MG patients. [file 9646209.f2.docx]

**Table S2. The anonymous data set of MuSK-MG patients**

| **Pts** | **Age at onset** | **Gender** | **Anti-MuSK abs**  **(nmol/L)** | **k free**  **(mg/L)** | **λ free**  **(mg/L)** | **k/λ** | **IgG1**  **(g/L)** | **IgG2**  **(g/L)** | **IgG3**  **(g/L)** | **IgG4**  **(g/L)** | **MGFA class at blood draw** | **Immunosuppressive**  **therapy** | **Dose of P**  **(mg)** |
| --- | --- | --- | --- | --- | --- | --- | --- | --- | --- | --- | --- | --- | --- |
| 1 | 17 | F | 0.09 | 13.90 | 13.40 | 1.04 | 8.19 | 5.58 | 1.20 | 0.28 | II b | P+RTX | 25 e/o/d |
| 2 | 13 | F | 1.10 | 42.70 | 18.40 | 2.32 | 9.29 | 7.15 | 1.05 | 1.00 | III b | none |  |
| 3 | 47 | M | 1.10 | 10.20 | 5.40 | 1.89 | 1.85 | 2.20 | 0.16 | 0.13 | III b | P | 35 e/o/d |
| 4 | 72 | M | 0.56 | 23.10 | 11.10 | 2.08 | 3.17 | 2.08 | 0.06 | 0.04 | III a | P+A | 37.5 e/o/d |
| 5 | 45 | F | 0.95 | 41.20 | 21.30 | 1.93 | 9.18 | 3.78 | 0.04 | 0.39 | III b | P | 20 e/o/d |
| 6 | 35 | F | 0.94 | 35.30 | 14.60 | 2.42 | 10.40 | 7.90 | 0.06 | 0.03 | IV b | P+Cy+ChE | 40 e/o/d |
| 7 | 62 | F | 0.99 | 4.00 | 4.60 | 0.87 | 1.58 | 0.20 | 0.06 | 0.03 | II b | P+3,4-DAP | 25 e/o/d |
| 8 | 51 | F | 0.77 | 46.70 | 27.20 | 1.72 | 7.61 | 7.50 | 1.10 | 0.84 | III b | P+Cy+ChE | 25 e/o/d |
| 9 | 63 | F | 1.15 | 22.10 | 11.00 | 2.01 | 9.06 | 8.20 | 0.43 | 0.27 | IV b | P |  |
| 10 | 46 | F | 0.77 | 4.00 | 4.60 | 0.87 | 1.92 | 1.82 | 0.19 | 0.15 | III b | P+MMF | 35 e/o/d |
|  |  |  | 0.83 | 4.00 | 4.60 | 0.87 | 1.58 | 0.20 | 0.06 | 0.03 | III b | P+MMF+RTX | 25 e/o/d |
| 11 | 62 | F | 1.32 | 18.60 | 11.50 | 1.62 | 3.64 | 2.46 | 0.83 | 0.11 | III b | P+MMF | 37.5 e/o/d |
|  |  |  | 1.41 | 27.60 | 14.90 | 1.85 | 4.97 | 5.00 | 0.48 | 0.11 | II b | P+MMF+RTX | 17.5 e/o/d |
| 12 | 38 | M | 1.12 | 57.10 | 31.20 | 1.83 | 0.67 | 7.10 | 1.10 | 0.68 | III b | P+A | 25 e/o/d |
|  |  |  | 1.09 | 49.80 | 41.10 | 1.21 | 5.60 | 7.20 | 1.20 | 0.53 | III b | P+MMF+RTX | 17 e/o/d |
| 13 | 37 | F | 0.89 | 32.30 | 14.70 | 2.20 | 17.00 | 8.00 | 1.08 | 0.71 | IV b | P+MMF | 50 d |
|  |  |  | 0.49 | 13.50 | 7.50 | 1.80 | 2.99 | 1.95 | 0.48 | 0.14 | III b | P+MMF+RTX | 37 d |

Abbreviations: P=prednisone; e/o/d=every other day; d= daily; CY= cyclosporine; ChE= acetylcholinesterase inhibitors; 3,4-DAP=3,4-diaminopyridine; MMF= mycophenolate mofetil; RTX=rituximab.
